# Postural stability during standing and its association with physical and cognitive functions in non-dialysis chronic kidney disease patients

**DOI:** 10.1007/s11255-019-02192-4

**Published:** 2019-06-18

**Authors:** Thomas J. Wilkinson, Daniel G. D. Nixon, Alice C. Smith

**Affiliations:** 1 Leicester Kidney Lifestyle Team, Department of Health Sciences, University of Leicester, Leicester, LE1 7RH UK; 2 NIHR Leicester Biomedical Research Centre, Leicester, LE5 4PW UK

**Keywords:** Postural instability, Balance, Chronic kidney disease, Physical function, Cognitive function

## Abstract

**Purpose:**

Chronic kidney disease (CKD) is characterised by poor physical function. A possible factor may be aberrant changes to balance and postural stability (i.e. ability to maintain centre of pressure (COP)). Previous research has exclusively focused on patients undergoing renal replacement therapy (RRT). The current study investigated postural stability in a group of CKD patients not requiring RRT.

**Methods:**

30 CKD patients (aged 57.0 ± 17.8 years, 47% female, mean eGFR 42.9 ± 27.2 ml/kg/1.73 m^2^) underwent a series of physical function assessments including the sit-to-stand-5 and -60, incremental shuttle walk test, gait speed, and short physical performance battery. Postural stability (defined as total COP ellipse (mm^2^) displacement) was measured using the Fysiometer board. Control reference data were provided by the manufacture. Cognitive function was assessed using the ‘Montreal Cognitive Assessment-Basic’ (MOCA-B)’.

**Results:**

CKD patients had poorer postural stability during quiet standing than reference values across all age categories (≤ 39 years, 24.9 ± 11.3 vs. 10.4 ± 1.8 mm^2^; 40–59 years, 34.3 ± 19.0 vs. 17.7 ± 6.2 mm^2^; ≥ 60 years, 39.7 ± 21.2 vs. 16.8 ± 2.9 mm^2^, all comparisons *P* < 0.001). Reductions in postural stability were associated with both physical and cognitive functioning. In females only, postural stability worsened with declining renal function (*r* = − 0.790, *P* < 0.01).

**Conclusions:**

To our knowledge, this is the first and largest experimental report concerning measurement of postural stability of CKD patients not requiring RRT. Our findings suggest that postural stability is associated with worse physical and cognitive functioning in this patient group.

**Electronic supplementary material:**

The online version of this article (10.1007/s11255-019-02192-4) contains supplementary material, which is available to authorized users.

## Introduction

Chronic kidney disease (CKD) is characterised by poor physical function that concomitantly declines with disease progression [1]. In patients not requiring renal replacement therapy (RRT), along with reducing quality of life and the ability to conduct activities of daily living, poor physical functioning is a significant prognosticator of mortality, adverse clinical outcomes [2] and falls [3].

Centre of pressure (COP) is the point of application of the resultant force between the feet and ground [4]. Postural stability is defined as the body’s ability to maintain COP relative to the base of support, either in a fixed position or during movement such as standing and walking [5]. Previous research into postural stability in renal populations has exclusively focused on RRT patients. Shin et al. [6] reported that under static balance conditions, haemodialysis (HD) patients exhibited lower postural stability compared to healthy individuals, whilst Blake et al. [7] reported postural stability was 39% poorer in HD patients than controls. Similar findings have been observed in renal transplant recipients (RTRs) [8]. In HD patients, lower postural stability is associated with poor self-reported physical function [7], and is considered an important cause of falls in the elderly [8, 9]. No research has investigated postural stability in non-dialysis-dependent CKD patients.

Postural stability is the product of a complex interplay of the sensory information processing (i.e. visual, vestibular, and proprioceptive) and motor output. Impairment to these processes can lead to decreased control of posture [7, 8, 10]. In renal patients [6, 8] and the elderly [11], impairment to postural stability may be a result of muscle atrophy and weakness [6] which reduces proprioception efficiency [8]. Along with physical functioning, there is also evidence that mobility and balance are associated with cognitive processing [6, 10]. In HD patients, postural stability was reduced by the addition of the performance of a simultaneous cognitive task [6]. It is thought that postural stability could be reduced by impaired visuospatial function or visual memory [12].

Evaluating postural stability in patients is clinically important as it is a known risk factor for falls [8, 9], and associated with reductions in poor cognitive processing [6] and self-reported physical function [7]. Early identification of patients with poor postural stability may allow appropriate intervention. Given that postural stability is reduced in other renal populations, it appears important for further investigation into the prevalence of poor postural stability and factors associated with it in patients with non-dialysis-dependent CKD. The objectives of the current study were to: (1) investigate postural stability in CKD compared to control reference values; (2) explore the association of postural stability with physical function; (3) explore the relationship between postural stability and cognitive functioning; and (4) investigate potential variables that contribute to postural stability. We hypothesised that CKD patients would have significantly poorer postural stability that a control reference cohort, and that poor postural stability would be associated with reductions in physical and cognitive functioning.

## Materials and methods

This is a sub-analysis of postural stability data collected from CKD patients not requiring RRT as part of a larger cross-sectional study (registered prospectively as ISRCTN11615440) looking at cardiovascular risk and physical condition in kidney patients. Patients attended Leicester General Hospital, UK, for a single visit where outcome measures were taken.

### Participants

Patients attending nephrology outpatient clinics based at the Leicester General Hospital, UK, were recruited to take part. Eligible patients were aged ≥ 18 years with diagnosed CKD not requiring RRT. Patients were excluded if they were pregnant, or had difficulties that limited provision of informed consent. Ethical approval was sought from the NRES Committee East Midlands–Derby (14/EM/1049) and the study was conducted in accordance with the Helsinki Declaration. Informed consent was obtained from all individual participants included in the study.

### Outcomes

#### Postural stability

Postural stability was assessed using a FysioMeter device. This is a modified Nintendo Wii Balance board (Nintendo, Kyoto, Japan) that contains four transducers used to assess force distribution and resultant COP displacement. 16-bit digital data samples at approximately 100 Hz were transferred via Bluetooth technology to custom-made software on a portable computer (FysioMeter ApS, Brønderslev, Denmark). The data were filtered using a fourth-order Butterworth filter with a cut-off frequency of 20 Hz. The device has been validated against a laboratory-grade force platform (AMTI Model OR6-5, Watertown, USA) in tasks of varying difficulty [13] and used in a variety of clinical populations [9, 10].

**Fig. 1:**
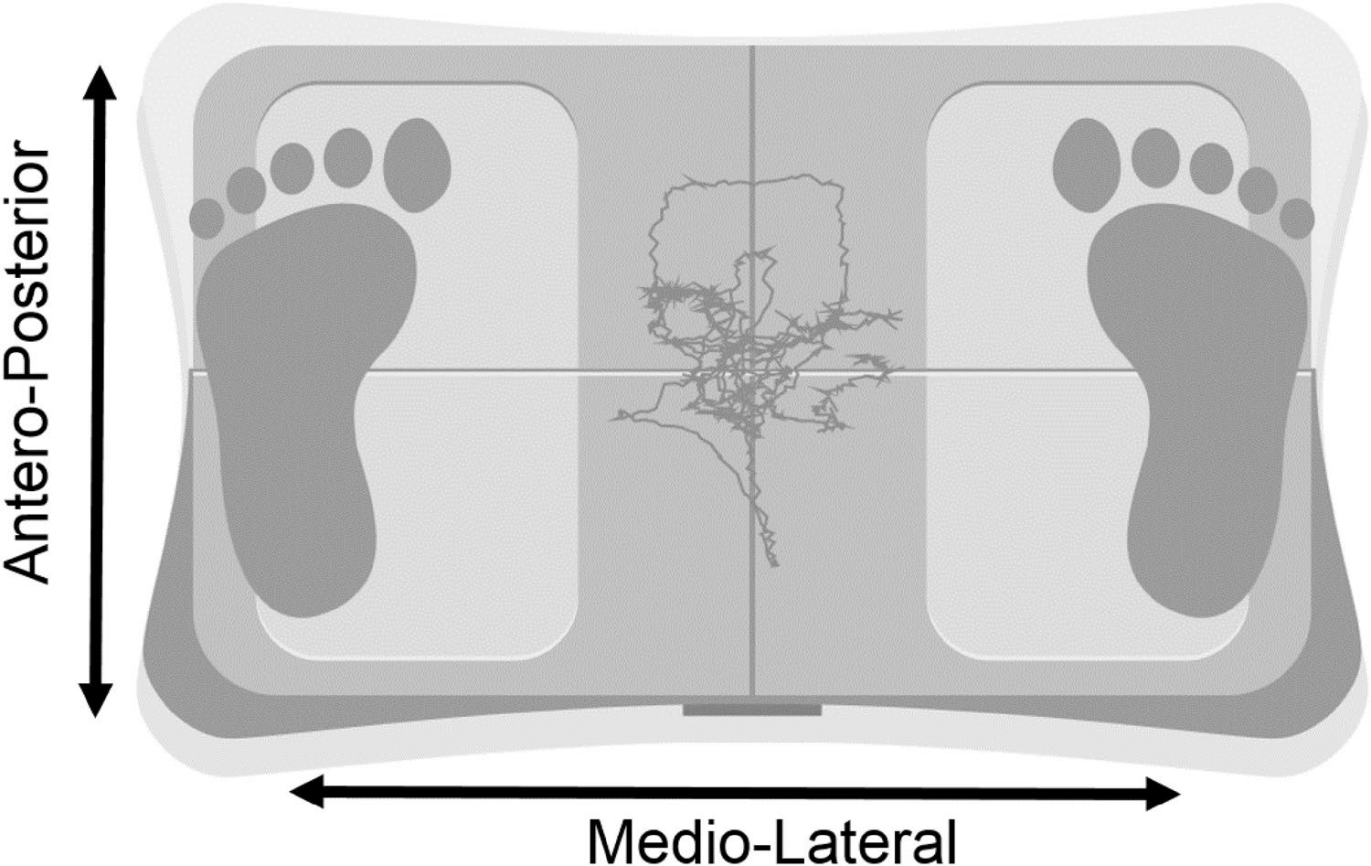
Fysiometer device and example trace for COP output. Red line shows changes in COP trace. Total COP ellipse area, representing the sum of postural sway in the anteroposterior and mediolateral directions, is calculated after 30 s of standing

Patients stood bipedal on the board with their feet shoulder-width apart and their eyes open. Patients were instructed to stand quietly for 30 s whilst keeping their head facing forward. Total COP ellipse area (mm^2^), representing the sum of postural sway in the anteroposterior and mediolateral directions, was tracked. The average of three attempts was taken with an inability to stand for the full duration resulting in no score. Greater body sway (i.e. poorer stability of COP) resulted in greater COP ellipse area. Figure 1 shows the device and COP ellipse area output.

Using the same protocol, comparative control reference data were taken from the normative values of 354 adults (aged 20–99 years) provided online by the manufacturers (FysioMeter ApS). Data for the reference cohort were divided into the pre-defined age categories for each gender. The average age of this cohort was 55.1 years (41% males). Full characteristics of this group are reported elsewhere [14].

#### Basic demographic and clinical information

Demographic (age, sex, ethnicity) and clinical (co-morbidity, renal function, latest haematology and blood chemistry counts, medication, disease aetiology) data were taken from medical records.

#### Physical function

To assess objective physical function, several tests were used:

- The ‘sit-to-stand’ (STS)-5 and -60 tests were used as measures of lower body strength and muscle endurance [15]. Sitting on a seat (43.2 cm from the ground) with their hands across their chest, patients were asked to: (1) perform five complete STS cycles as fast as possible (STS-5); and (2) perform as many STS cycles in 60 s (STS-60).
- Usual gait speed was measured over a marked 4-m course, with the faster of two trials used for analysis. Gait speed is a well-established predictor of mortality in CKD [2]. A gait speed ≤ 0.8 m/s was deemed as poor due to its association with increased mortality in this population [2].
- The ‘Incremental Shuttle Walk Test’ (ISWT) was used to measure cardiorespiratory walking and exercise capacity. The ISWT is a symptom-limited maximally progressive test that involves the patient walking a total of 10 m back and forth, and around two cones. Walking pace, through a series of bleeps, is externally dictated [16]. We have recently validated this test in CKD [17].
- The ‘Short Physical Performance Battery’ (SPPB) is a well-established measure of lower extremity function, previously used in CKD research [18]. A total score (/12) is derived from: (1) usual 4 m gait speed; (2) tests of standing balance (side-by-side, semi-tandem, tandem positions); and (3) STS-5. A five-level categorical score was assigned to each test; 0 representing inability to complete the test and 4 representing the highest level of performance [19]. A total SPPB score < 10 was deemed as poor due to its association with increased mortality in this population [2].

#### Cognitive function

The ‘Montreal Cognitive Assessment-Basic’ (MOCA-B) was used to test cognitive impairment. A validated 30-point test, the MOCA-B evaluates six cognitive domains: visual perception (superimposed objects), executive functioning (alternating trail making; word similarity; problem-solving), language (fruit fluency; animal naming), attention (modified digit Stroop), memory (five-word recall), and orientation (time and place) [20].

#### Body composition

To assess the effect of body composition, we measured skeletal muscle mass and body fat using multi-frequency bioelectrical impedance analysis (InBody 370, CA, USA). Both absolute (kg) and relative (% of total body mass) body composition values were calculated. Our group has validated this device in a cohort of non-dialysis-dependent CKD patients and RTRs [21].

### Statistical analysis

Demographic data are shown as mean (± SD) or *n* (%). As per manufacture settings, control reference data were categorised into the age ranges ≤ 39 (*n *= 103), 40–59 (*n *= 87), ≥ 60 (*n *= 164). Independent samples *T* tests were used to investigate the differences between CKD and reference data. The difference between CKD and reference data is expressed as a % of the CKD data. Distribution of COP was normally distributed (assessed using Shapiro–Wilk test) across three age categories (≤ 39 years, 40–59 years, > 60 years) in the CKD group.

Bivariate and partial correlations were used to explore the association between postural stability, and physical and cognitive function. For all associations, an unadjusted bivariate analysis (Model 1) and partial correlation analysis adjusted for age, sex, ethnicity, BMI, and eGFR (Model 2) were performed. Potential predictive factors of postural instability were analysed using forced entry linear regression modelling. Regression models were run using both absolute and relative body composition variables. The number of missing data is shown as supplementary material 1. Statistical significance was recognised as *P* < 0.05. Data analysis was performed on IBM SPSS Statistics 24 (NY, USA) and Prism 7 (CA, USA).

## Results

Basic characteristics of patients consented to the study are shown in Table 1. Overall, the mean age was 57.0 ± 17.8 years old with 47% of the cohort female. The mean eGFR was 42.9 ± 27.2 [min: 8, max: 90] ml/kg/1.73 m^2^ with the majority of patients (74%) in CKD stages 3a–5. No patients required RRT. The mean age and gender distribution was similar to the control reference cohort. The functional status of our CKD sample was poor with eight (27%) patients recording a SPPB score < 10 and two (7%) patients having a gait speed ≤ 0.8 m/s.

**Table 1:** Patient characteristics

	*n* = 30
Age (years)	57.0 ± 17.8
Sex, *n* female (%)	14 (47)
Height (cm)	170.4 ± 8.2
Body mass (kg)	86.0 ± 16.5
Body mass index (kg/m^2^)	29.5 ± 4.8
Body fat (%)	37.5 ± 5.3
Waist circumference (cm)	101.3 ± 13.8
Hip circumference (cm)	104.6 ± 31.5
Hip to waist ratio	1.3 ± 1.7
Ethnicity	
White British, *n* (%)	24 (80)
Asian, *n* (%)	5 (17)
Caribbean, *n* (%)	1 (3)
Clinical parameters	
Systolic blood pressure (mmHg)	133 ± 15
Diastolic blood pressure (mmHg)	80 ± 13
eGFR (ml/kg/1.72 m^2^)	42.9 ± 27.2
Haemoglobin (g/dl)	12.9 ± 1.6
Disease aetiology	
Diabetic nephropathy, *n* (%)	5 (17)
IgA nephropathy, *n* (%)	5 (17)
Polycystic kidney disease, *n* (%)	3 (10)
Primal focal segmental glomerulosclerosis, *n* (%)	2 (7)
Other, *n* (%)	4 (12)
Unknown/not stated, *n* (%)	11 (37)
Co-morbidities	
Diabetes, *n* (%)	9 (30)
(Diagnosis of) hypertension, *n* (%)	21 (70)

Unless stated otherwise, data presented as mean (± SD)

*eGFR* estimated glomerular filtration rate

## Postural stability in CKD versus control reference data

**Fig. 2:**
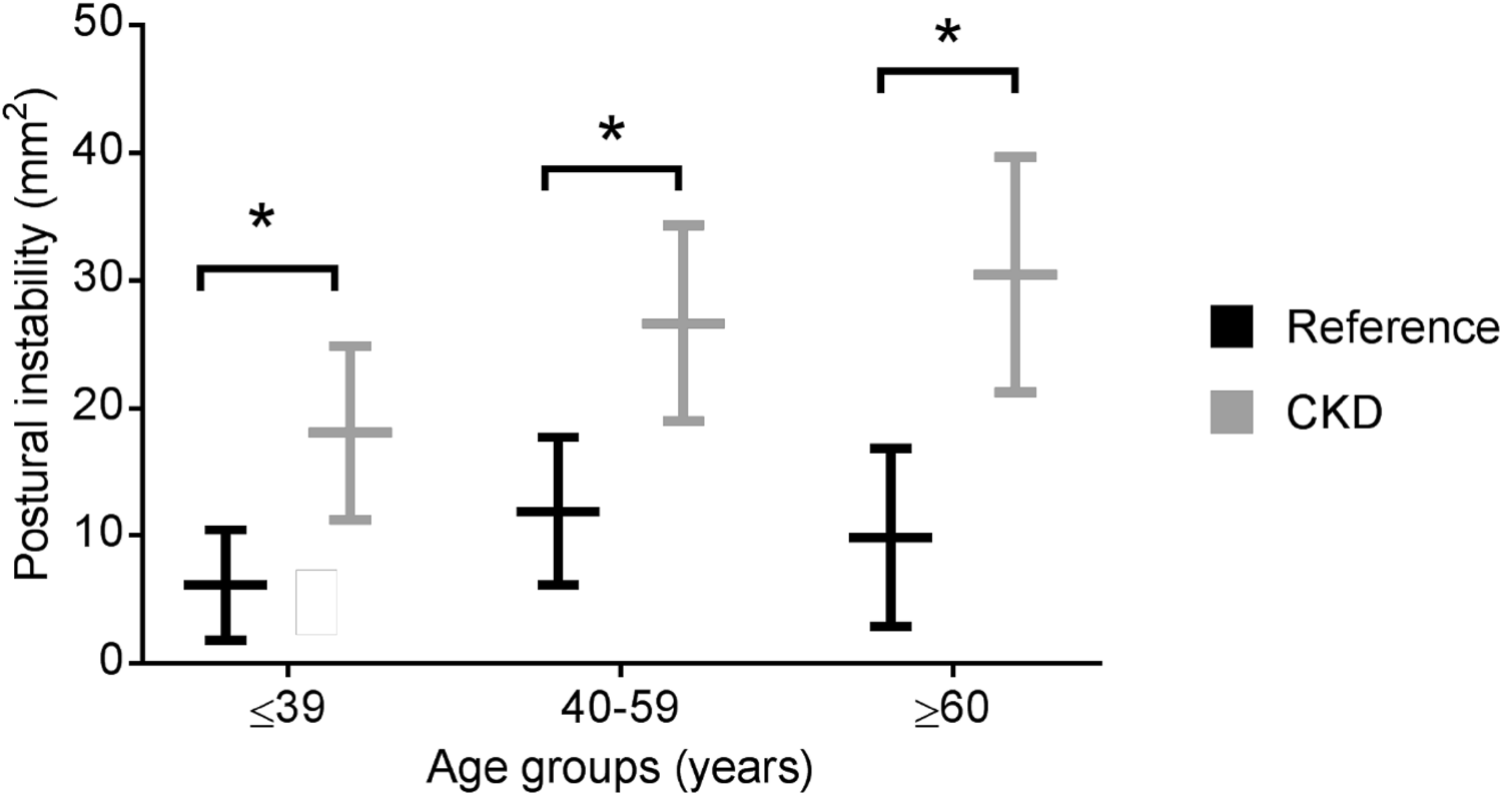
Differences in postural stability between CKD patients and control reference data. Postural instability defined as using total COP ellipse area (mm^2^), representing the sum of postural sway in the anteroposterior and mediolateral directions, over 30 s. *CKD* chronic kidney disease

Postural stability was significantly lower in CKD than the reference cohort across all age ranges (Fig. 2). The mean COP ellipse area for CKD patients aged ≤ 39 years was 24.9 ± 11.3 mm^2^ compared to 10.4 ± 1.8 mm^2^ for the reference cohort (− 58%, *P *< 0.001). In CKD patients aged 40–59 years, the mean COP ellipse area was 34.3 ± 19.0 mm^2^ compared to 17.7 ± 6.2 mm^2^ for the reference cohort (− 48%, *P *< 0.001), and the mean COP ellipse area for CKD patients ≥ 60 years was 39.7 ± 21.2 mm^2^ compared to 16.8 ± 2.9 mm^2^ for the reference cohort (− 58%, *P *< 0.001). There was no statistically significant difference between CKD patients aged ≤ 39 and ≥ 60 years (*P *= 0.12), or those aged 40–59 and ≥ 60 (*P *= 0.57). CKD patients aged ≤ 39 had 33% poorer postural stability than the reference cohort aged ≥ 60 years (*P *< 0.001).

## Postural stability and physical functioning

Table 2 shows the association between postural stability and physical function. In an unadjusted model and adjusted model 2 (controlling for age, sex, ethnicity, BMI, and eGFR), reductions in postural stability were significantly associated with poorer physical function scores (all tests). Reductions in postural stability were strongly associated with an increased time to complete the STS-5 test (*r *= 0.846, 0.869, *P* < 0.01 across all models).

**Table 2:** The association of postural stability and physical and cognitive function

	Model 1 (unadjusted)		Model 2 (unadjusted)	
	***r***	*P*	*r*	*P*
STS-5 (s)	0.846	< 0.01*	0.869	< 0.01*
STS-60 (repetitions)	− 0.481	0.01*	− 0.500	0.04*
Gait speed (m/s)	0.624	< 0.01*	− 0.500	0.04*
ISWT (m)	− 0.622	< 0.01*	− 0.488	0.05*
SPPB (total score)	− 0.707	< 0.01*	− 0.674	0.03*
MOCA-B (total score)	− 0.770	< 0.01*	− 0.723	0.01*

Significant values are in bold

Model 2 = adjusted for age, sex, ethnicity, BMI, and eGFR

*eGFR* estimated glomerular filtration rate, *BMI* body mass index, *STS* sit-to-stand, *ISWT* incremental shuttle walk test, *SPPB* short physical performance battery, *MOCA-B* Montreal Cognitive Assessment-Basic. Significance set at < 0.05

## Postural stability and cognitive function

The average MOCA-B score was 27.6 ± 2.8 with 29% of patients scoring the maximum of 30 points. Poorer postural stability was strongly correlated with a lower MOCA-B score (Model 1: *r *= − 0.770, *P *< 0.01; Model 2: *r *= − 0.723, *P *= 0.01) (Table 2).

## Factors influencing postural instability

**Fig. 3:**
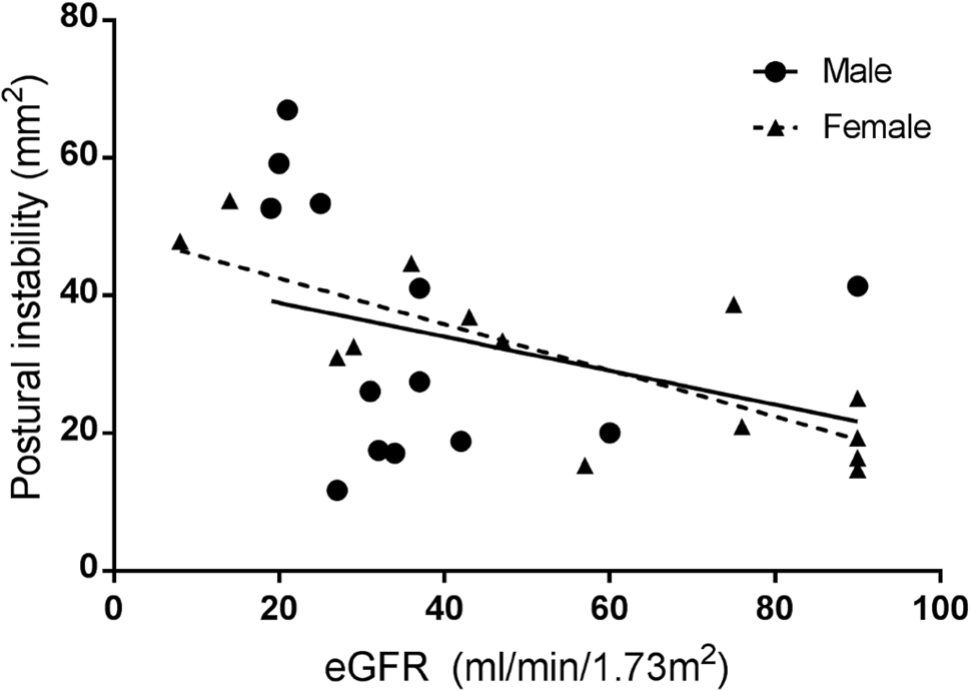
Relationship between renal function and postural instability in male and female CKD patients. Postural instability defined as using total COP ellipse area (mm^2^), representing the sum of postural sway in the anteroposterior and mediolateral directions, over 30 s. *CKD* chronic kidney disease, *eGFR* estimated glomerular filtration rate

Postural stability worsened with declining renal function (eGFR), although this was statistically significant in female patients only (male, *r *= 0.259, *P *= 0.39; female, *r *= 0.790, *P *< 0.01) (Fig. 3). The only significant predictor variable of postural stability was greater absolute skeletal muscle mass (*β* = 1.301, *P *= 0.03). Relative muscle was not significant predictors of postural stability (Table 3).

**Table 3:** Potential predictive factors of postural stability in male and female CKD patients

	*B*	95% CI	β	*t*	*P*
Absolute body composition					
Age (years)	0.038	− 1.022 to 1.098	0.017	0.075	0.94
Sex	17.552	− 24.878 to 59.982	0.219	0.858	0.40
eGFR (ml/kg/1.72 m^2^)	− 0.466	− 1.221 to 0.289	− 0.306	− 1.279	0.21
Skeletal muscle mass (kg)	3.542	0.314 to 6.771	0.570	2.275	0.03*
Body fat (kg)	− 0.527	− 2.061 to 1.006	− 0.163	− 0.713	0.48
Relative body composition					
Age (years)	0.241	− 1.035 to 1.517	0.105	0.391	0.70
Sex	− 10.059	− 49.451 to 29.333	− 0.126	− 0.530	0.60
eGFR (ml/kg/1.72 m^2^)	− 0.425	− 1.262 to 0.411	− 0.279	− 1.055	0.30
Skeletal muscle mass (%)	− 1.945	− 20.678 to 16.788	− 0.185	− 0.215	0.83
Body fat (%)	− 0.858	− 12.363 to 10.647	− 0.131	− 0.155	0.88

Significant values are in bold

*eGFR* estimated glomerular filtration rate. Significance set at < 0.05

## Discussion

The primary findings of the current study were that: (1) CKD patients have poorer postural stability than a control reference cohort; (2) reduced postural stability is associated with poor physical and cognitive functioning; and (3) postural stability worsens with declining renal function. To our knowledge, this is the first experimental report concerning measurement of postural stability in this population.

Using total COP ellipse area during a 30-s quiet standing task as measure of postural stability, we found that patients exhibited significantly greater displacement than a control reference cohort. On average, the postural stability was between 48% and 58% poorer than the reference values. In 19 patients undergoing HD treatment, Shin et al. [6] showed that during quiet standing, total sway area was 88% larger than healthy controls. In that study, anteroposterior displacement was 38% larger and mediolateral displacement 54% greater. Blake et al. [7] showed that anteroposterior displacement was 39% greater in 12 patients undergoing HD than healthy controls. Here, postural sway was measured over a 10-s period with patients demonstrating impairments under eyes open and closed conditions. Interestingly, balance deteriorated more than controls when visual input was eliminated and greater reliance was placed on the somatosensory and vestibular systems; the authors proposed that elevated postural sway was a result of proprioceptive dysfunction. In 19 RTRs, Zanotto et al. [8] found that during 30 s of quiet standing, RTRs had greater anteroposterior displacement (22%) than healthy controls. The authors postulated that increased postural sway may indicate an impaired capacity to rely on proprioceptive and vestibular information. Unfortunately, no data relating to proprioceptive function were recorded in our current study.

Reduced postural stability was associated with poor physical function in our group. In particular, postural stability was strongly associated with the time taken to complete the STS-5 and SPPB test. Given the importance of balance in standing from a seated position, this result is perhaps expectable. This suggests that efforts to improve postural stability may be a key factor in improving physical function in this group. Research into the relationship between postural stability and physical function is limited. In HD patients, greater COP displacement was associated with poor self-reported physical function as measured using the SF-36 questionnaire [7]. In patients with osteoarthritis, decreased postural control (assessed using a one-leg stand test) was associated with a longer time in the ‘Get Up and Go’ test [22], and in healthy middle-aged participants, greater anteroposterior body sway was associated with poorer gait speed [23].

We identified a strong significant relationship between postural stability and cognitive function as measured by the MOCA-B. The MOCA-B is a validated measure of cognitive impairment and includes assessment of visual perception, executive functioning, and memory. It has been suggested that postural stability could be reduced by impaired visuospatial function or visual memory [12]. Murakami et al. [24] showed in Parkinson’s disease patients, poor postural stability (measured by the Unified Parkinson’s Disease Rating Scale) was associated with reduced MOCA scores, particularly the visuospatial and orientation subdomains. It is thought that the postural control system integrates sensory information and produces motor commands in response to environmental changes [24]. This level of central motor processing also includes visuospatial and executive functions, and motor and cognitive impairments may share a common pathophysiology (e.g. neural pathways) [6, 24]. With the MOCA-B acting as a ‘proxy’ indicator of visuospatial and executive function, which is important in posture [12], this may explain the association in our data.

Previous research has suggested that in renal patients [6, 8] and the elderly [11], reductions in postural stability may be the result of aberrant body composition. Unlike previous work [6, 8, 25], we found no association between poor postural stability and increased fat mass. However, we did observe reduced postural stability in those with greater absolute muscle mass. This contrasts research by Zanotto et al. [8] who postulated that muscle atrophy reduced proprioception efficiency. Adjusting muscle mass relative to total body mass removed any association with postural stability suggesting that it may be the actual load of the body, rather that the composition of muscle and fat, that is important. Indeed, an exploratory analysis revealed a significant correlation between body mass and postural instability (*r *= 0.47, *P *= 0.01). Body mass has been shown previously to positively correlate with postural sway and obesity is associated with increased postural instability [25, 26]. In our sample, it appears that the majority of this ‘mass’ comes from the skeletal muscle. Alonso et al. [26] suggested that greater muscle mass and a smaller support base increase sway area, and that rather than a worsening of balance may be strategy used to stabilize COP. However, although not observed in our sample, the influence of extra fat mass on increased biomechanical loadings (e.g. joint torques and muscle forces, body segment inertial parameters, sensorimotor and central nervous systems dysfunction [25]) should not be discounted. Our data suggest that reducing body mass may confer favourable effects on COP and, therefore, physical function.

Given the importance of postural stability in CKD, efforts should be made to measure it appropriately. Postural stability is preferably investigated using posturography [9, 13]. However, such measures are often costly, problematic to locate, and require experienced personnel; therefore, this form of assessment is not often feasible. Consequently, subjective assessment, such as the Berg Balance Scale, is commonly used in CKD [27, 28]. While more feasible, they are limited by ceiling effects and an inability to distinguish small changes [29], as demonstrated in exercise trials in CKD [28]. In our current study, we used a modified Nintendo Wii Balance board (FysioMeter device) to assess COP displacement. A validated assessment of posture control [13], the FysioMeter costs a small fraction of a laboratory-grade force platform, is mass-marketed, and is portable. Accordingly, it has the potential to become a key component in physical functional assessment [10, 13]. If reduced postural stability is detected in patients, efforts should be made to improve it. Whilst the contributing factors are complex and likely distinct to each individual, forms of specific-balance training and exercise therapy (e.g. unilateral standing) have been shown an effective modality in the rehabilitation of balance deficits in patients with various conditions (e.g. chronic ankle instability [29], older adults [30], and Parkinson’s disease [31]).

Some limitations of this study should be acknowledged. First, our assessment of postural stability occurred during a single task (quiet bipedal standing, eyes open, for 30 s). It would have been advantageous to assess posture during different scenarios (e.g. dual task (i.e. addition of a cognitive task) or eyes closed to remove visual information). However, it is important to recall that the large deficits seen were observed using a simple standing task; worryingly, the addition of dual tasks or removal of sensory input may exacerbate this impairment further. Second, the Fysiometer device is unable to differentiate variation in anteroposterior and mediolateral directions. However, given the strong associations with total COP ellipse area and the ease of use of the device, it is unlikely on a practical level that this detail of information is needed by healthcare professionals. Third, our study has a low sample size and as a subset-analysis of a larger study, no a priori sample size was calculated. However, our sample of *n *= 30 patients is greater than previous studies investigating postural instability in renal populations (e.g. HD, *n *= 19 [6], *n *= 12 [7], RTRs, *n *= 19 [8]). Whilst no patients in the current study had evidence of peripheral neuropathy or neuropathic conditions, this may be an important consideration in further study of postural stability in this patient group. Although our analysis is further limited in its cross-sectional data, it does provide evidence showing postural stability is associated with both physical and cognitive functioning.

## Conclusion

In conclusion, CKD patients not requiring RRT have reduced postural stability during quiet standing. We have shown that this reduction in postural stability is associated with poor physical, particularly those involving standing and cognitive functioning, and postural stability worsened along with declining renal function. These findings reveal that postural stability has potential clinical implications on patient physical and cognitive functioning. Whilst it is unlikely to be routinely assessed in all patients, our data reveal that, irrespective of age, postural stability assessments should be considered in those with advancing disease, increased body mass, and those with reduced physical functioning. Further research is needed to examine the mechanisms behind postural stability, and interventions that can be used to improve it.

## Electronic supplementary material

Below is the link to the electronic supplementary material.

Supplementary material 1 (DOCX 16 kb)
