# Supplementary material for: Postural stability during standing and its association with physical and cognitive functions in non-dialysis chronic kidney disease patients
Source: Int Urol Nephrol. 2019 Jun 18;51(8):1407–14. doi: 10.1007/s11255-019-02192-4 (PMC6660492; doi:10.1007/s11255-019-02192-4)
Supplement: Supplementary file 1 — Supplementary material 1 (DOCX 16 kb) [file 11255_2019_2192_MOESM1_ESM.docx]

Supplementary material 1. Number of missing data

|  | *n* (%) missing |
| --- | --- |
|  |  |
| Age | 0 (0%) |
| Sex | 0 (0%) |
| Ethnicity | 0 (0%) |
|  |  |
| *Body composition and anthropometry* |  |
| Height | 0 (0%) |
| Body mass | 0 (0%) |
| Body mass index | 0 (0%) |
| Waist circumference | 2 (7%) |
| Hip circumference | 2 (7%) |
| Hip to Waist ratio | 2 (7%) |
| Skeletal muscle mass | 0 (0%) |
| Body fat | 0 (0%) |
| Skeletal muscle mass | 0 (0%) |
| Body fat | 0 (0%) |
|  |  |
| *Clinical parameters* |  |
| Systolic blood pressure | 0 (0%) |
| Diastolic blood pressure | 0 (0%) |
| eGFR | 0 (0%) |
| Hemoglobin | 1 (3%) |
| Disease aetiology | 0 (0%) |
| Co-morbidities (hypertension and diabetes record) | 0 (0%) |
|  |  |
| Postural stability (COP ellipse area) | 2 (7%) |
|  |  |
| *Physical function* |  |
| STS-5 | 0 (0%) |
| STS-60 | 0 (0%) |
| Gait speed | 0 (0%) |
| ISWT | 1 (3%) |
| SPPB | 0 (0%) |
|  |  |
| *Cognitive function* |  |
| MOCA-B | 6 (20%) |
|  |  |

Unless stated otherwise, data presented as mean (±SD). eGFR = Estimated glomerular filtration rate; COP = Centre of pressure; STS = Sit-to-stand; ISWT = Incremental shuttle walk test; SPPB = Short physical performance battery; MOCA-B = Montreal Cognitive Assessment-Basic
